# Characterization of the Intergenic Spacer rDNAs of Two Pig Nodule Worms, *Oesophagostomum dentatum* and *O. quadrispinulatum*

**DOI:** 10.1155/2014/147963

**Published:** 2014-08-13

**Authors:** Rui-Qing Lin, Li Shu, Guang-Hui Zhao, Tian Cheng, Shang-Shu Zou, Yuan Zhang, Ya-Biao Weng

**Affiliations:** ^1^Laboratory of Parasitology, College of Veterinary Medicine, South China Agricultural University, Guangzhou, Guangdong Province 510642, China; ^2^Yong Nian Veterinary Station, Fushun, Sichuan 643200, China; ^3^College of Veterinary Medicine, Northwest A&F University, Yangling, Shaanxi 712100, China

## Abstract

The characteristics of the intergenic spacer rDNAs (IGS rDNAs) of *Oesophagostomum dentatum* and *O. quadrispinulatum* isolated from pigs in different geographical locations in Mainland China were determined, and the phylogenetic relationships of the two species were reconstructed using the IGS rDNA sequences. The organization of the IGS rDNA sequences was similar to their organization in other eukaryotes. The 28S-18S IGS rDNA sequences of both *O. dentatum* and *O. quadrispinulatum* were found to have variable lengths, that is, 759–762 bp and 937–1128 bp, respectively. All of the sequences contained direct repeats and inverted repeats. The length polymorphisms were related to the different numbers and organization of repetitive elements. Different types and numbers of repeats were found between the two pig nodule species, and two IGS structures were found within *O. quadrispinulatum*. Phylogenetic analysis showed that all *O. dentatum* isolates were clustered into one clade, but *O. quadrispinulatum* isolates from different origins were grouped into two distinct clusters. These results suggested independent species and the existence of genotypes or subspecies within pig nodule worms. Different types and numbers of repeats and IGS rDNA structures could serve as potential markers for differentiating these two species of pig nodule worms.

## 1. Introduction 

The ribosome has been identified as a central hub for sensing the nature of a nascent protein chain, recruiting protein folding and translocation components, and integrating mRNA and nascent chain quality control [1]. Ribosomal RNA (rRNA) typically accounts for approximately 40% of all transcription within a cell, and ribosomal RNA comprises as much as 80% of the cellular RNA [2]. The rRNA transcripts, including 18S, 28S, and 5.8S rRNA, mature through the excision of their tandem spacer regions, for example, internal transcribed spacers (ITSs) and intergenic spacers (IGSs) [3]. The IGSs are biologically significant. Furthermore, the cell is capable of both regulating rRNA synthesis and sequestering large numbers of proteins to modulate essential molecular networks through the timely induction of various ribosomal IGS noncoding RNA (IGS RNA) transcripts [4].

IGS regions are composed of an extraordinary variety of repeats and RNA polymerase promoters and enhancers, which cause considerable inter- and intraspecific variations in parasites [5]. These variations make IGS rDNA a suitable marker for inferring evolutionary relationships among more closely related species and among strains within the same species [6] as well as for developing molecular detection approaches for infectious diseases [7–12]. The IGS rDNA regions of* Trypanosoma* [13],* Giardia* [8],* Leishmania* [6],* Toxoplasma gondii* and* Neospora caninum* [14],* Schistosoma haematobium*,* S. intercalatum,* and* S. mansoni* [15], and* S. japonicum* [5] have been studied and showed some organizational features common to the majority of eukaryotes.

Oesophagostomiasis, which is caused by nodular worms (*Oesophagostomum* spp.) and is commonly observed in pigs, ruminants, and primates (including humans), is often neglected by researchers and practitioners due to its mild symptoms [16]. However, severe infections can lead to significant socioeconomic problems and serious public health concerns [17–19]. Of the* Oesophagostomum* spp.,* O. dentatum* and* O. quadrispinulatum* have been identified as the two main causative agents of oesophagostomiasis in pigs [20, 21]. Moreover,* O. dentatum* was proposed as a potential model for genomic studies of strongylid nematodes [22]. The objective of this study was to determine the characteristics of the IGS rDNA regions of* O. dentatum* and* O. quadrispinulatum* collected from pigs at different geographical locations in Mainland China.

## 2. Materials and Methods

### 2.1. Parasite Samples

Isolates of* O. dentatum* (14 isolates) and* O. quadrispinulatum* (12 isolates) were collected from pigs at six geographical origins in Mainland China. Their codes, geographical origins, and accession numbers are listed in Table 1. Each adult parasite was washed extensively in physiological saline and was preliminarily identified at the species level based on its morphological characteristics [23].

### 2.2. DNA Isolation and PCR Amplification

Genomic DNA (gDNA) was extracted from individual adult worms through sodium dodecyl-sulfate/proteinase K treatment, column-purified using the Wizard SV Genomic DNA Purification System (Promega) and eluted with 40 *μ*L of H_2_O according to the manufacturer's recommendations. The DNA samples were then identified at the species level based on their ITS rDNA sequences [20] and stored at −20°C until further analysis.

The 28S-18S IGS rDNA sequences of* O. dentatum* and* O. quadrispinulatum* were amplified using PCR with the O28 (5′-ACGACATGTATACTGGTCAAGG-3′, forward) and O18 (5′-GCTTTGGTGCATGTATTAGCTC-3′, reverse) primers. The PCR reactions included 3 mM MgCl_2_, 0.5 *μ*M of each primer, 2.5 *μ*L of Ex Taq buffer, 0.2 mM of each deoxyribonucleotide, 0.5 U of Ex Taq DNA polymerase (TAKARA), 1 *μ*L of DNA sample, and double-distilled water to a total volume of 25 *μ*L. The procedures were performed in a thermocycler (Biometra) under the following conditions: 94°C for 5 min (initial denaturation), followed by 35 cycles of 94°C for 30 s (denaturation), 55°C for 1 min (annealing), 72°C for 45 s (extension), and a final extension at 72°C for 5 min. An aliquot (5 *μ*L) of each amplicon was examined on 1.0% agarose-TBE gels, stained with ethidium bromide (EB) and photographed upon transillumination. The DL2000 marker (TAKARA) was used to estimate the sizes of the IGS+ rDNA amplicons.

### 2.3. Purification, Cloning, and Sequencing of the IGS PCR Products

Representative PCR products were purified using spin columns (Wizard PCR-Prep DNA Purification System, Promega), and the purified PCR products were ligated into the pGEM-T easy plasmid vector (Promega) according to the manufacturer's recommendations. The recombinant plasmid was then transformed into* Escherichia coli* JM109 competent cells (Promega), and positive transformants containing recombinant plasmids were selected by PCR amplification. Cell cultures with confirmed recombinant plasmids were sent to Shanghai sangon Biological Engineering Biotechnology Company for sequencing using an ABI 377 automated DNA sequencer (BigDye Terminator Chemistry).

### 2.4. Sequence Analysis and Reconstruction of Phylogenetic Relationships

The characteristics of the 28S-18S IGS rDNA regions of* O. dentatum* and* O. quadrispinulatum* were determined by comparing these sequences with the previously published IGS rDNA sequences of* Skrjabingylus chitwoodorum* (AY295819),* Cylicocyclus nassatus* (AJ223348), and* Cyathostomum catinatum* (AJ223339); the 18S rDNA sequences of* Chabertia ovina* (AJ920341) and* Labiostrongylus bipapillosus* (AJ920337); and the 28S rDNA sequences of* Chabertia ovina* (AM039733) and* Labiostrongylus bipapillosus* (AJ512837).

The palindrome in EMBOSS 6.3.1 [24] (http://mobyle.pasteur.fr/cgi-bin/portal.py?#forms::palindrome) was used to identify inverted repeats in the* Oesophagostomum* species. Direct repeats were identified using REPFIND [25] at http://cagt.bu.edu/page/REPFIND_submit and Tandem Repeats Finder [26] at http://tandem.bu.edu/trf/trf.html. These repeats were identified with the criteria of nuclear match ≥10 bp and mismatch ≤1.

The phylogenetic relationships of the* O. dentatum* and* O. quadrispinulatum* isolates from the different geographical origins were reconstructed based on their IGS rDNA sequences using the neighbor-joining (NJ) method within the Mega 4.0 software and the Kimura 2-parameter model [27]. Phylograms were drawn using the TreeView program, version 1.65 [28].

## 3. Results and Discussion

**Figure 1:**
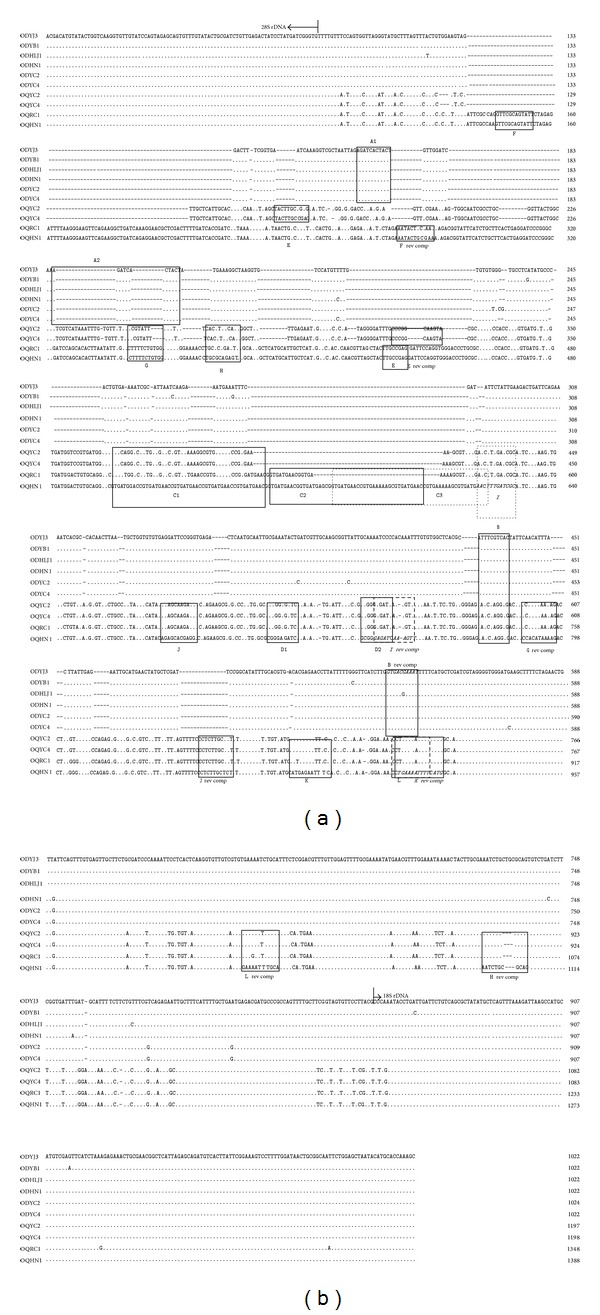
The alignment of the representative sequences of intergenic spacer rDNA (IGS rDNA) regions of* Oesophagostomum dentatum* and* O. quadrispinulatum*. Portions of the flanking 28S and 18S genes are shown. Dots (*·*) denote sequence identity to the first sequence. Dashes (-) represent nucleotide deletions. Motifs for two nematodes are boxed with a solid (or broken) line. See the text for additional details.

The 28S-18S IGS rDNA sequences of* O. dentatum* and* O. quadrispinulatum* from Mainland China had dynamic and highly complex structures. The first indication of this finding became apparent upon amplification of the IGS rDNA, which presented variable lengths ranging from 1000 to 1400 bp (data not shown). After removal of the flanking 28S and 18S rDNA sequences, the lengths of the IGS rDNA sequences were 759–762 bp and 937–1128 bp for* O. dentatum* and* O. quadrispinulatum*, respectively. The IGS of* O. dentatum* only contained 2 copies of one 11-nt direct repeat (A1 and A2) and one 10-nt inverted repeat (B and B rev comp). The IGS rDNA sequences of* O. quadrispinulatum* could be grouped into two types based on their lengths and characteristics. The longest, the IGS of OQHN1, exhibited the following features: (1) three complete 49-nt copies of direct repeat C beginning 409 nt downstream of the 5′ end of the IGS rDNA; (2) two copies of direct repeat D beginning 89 nt downstream of the last copy of direct repeat C; (3) one complete inverted repeat I; and (4) six short, incomplete inverted repeats (inverted repeats F, G, H, J, K, and L). Compared with OQHN1, the shortest sequence, OQYC4, showed the following features: (1) a new, incomplete inverted repeat E and (2) no repeats including two copies of direct repeat C, repeat F, repeat G, or inverted repeat F (Figure 1).

Eukaryotic ribosomes are very important for protein synthesis, cellular growth, and organismal development [2]. In most eukaryotes, rDNA is arranged in tandemly repeated units containing genes for the 18S, 5.8S, and 28S rRNAs, which are separated by spacers. The large intergenic spacer (IGS; formerly NTS) separating the 28S and 18S genes is internally repetitious: each repeat contains a tandem array of short subrepeat units [29, 30]. Alterations in these repeats mostly occur due to unequal crossing over during both sexual and asexual reproduction or in somatic cell lineages [31], and variations in the number of repeat units and, consequently, in the copy number of the regulatory elements can lead to the polymorphic lengths and structures observed in the IGS rDNA [2]. In this study, the IGS rDNA sequences of* O. dentatum* and* O. quadrispinulatum* were found to contain many short, direct, and inverted repeats, and different types and numbers of repeats were found both between the two pig nodule species and within* O. quadrispinulatum*. These differences suggested that these species are independent and that genotypes or subspecies exist within the known species of pig nodule worms.

Homologous chromosome pairing precedes meiotic recombination and may initiate without strand breakage by way of “kissing” interactions between the loops of extruded stem-loop structures [32]. Such recombination may be responsible for the existence of numerous repeats such as “cross-over hot-spot” (Chi) sequences [32]. The Chi site, which contains sequences of 5′-GCTGGTGG-3′ in the template strand and 5′-CCACCAGC-3′ in the complementary strand, has been found in the repetitive sequences within the first internal transcribed spacer of the rDNA of schistosomes [33]. Although these two sequences were not found in the IGS rDNA sequences of the two pig nodule worms examined in this study, two similar sites, with sequences of 5′-GCTGGTGT-3′ 93 bp upstream of the 5′ end of direct repeat B and of 5′-CCTGGCGG-3′ 9 bp downstream of the 3′ end of inverted repeat I, were found in the IGS rDNA sequences of* O. dentatum* and* O. quadrispinulatum*, respectively. These results indicated that the IGS rDNA might participate in homologous chromosome pairing.

**Figure 2:**
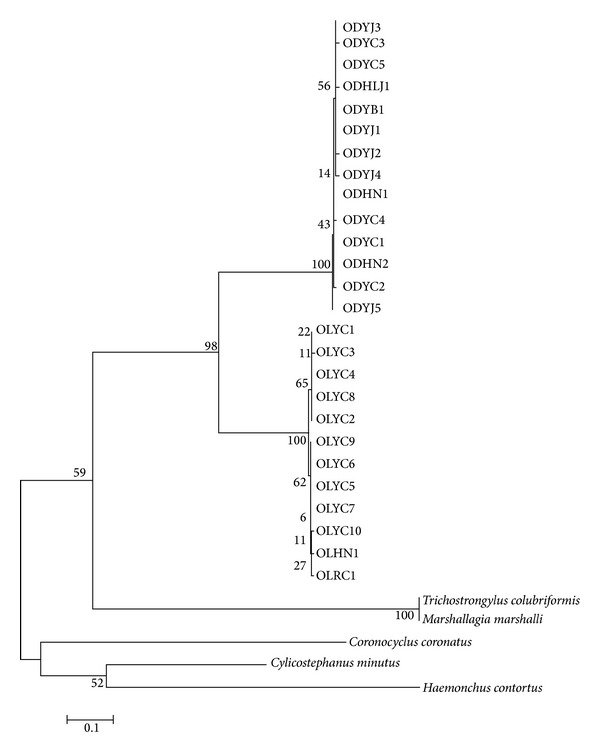
Phylogenetic relationships of* Oesophagostomum* and* O. quadrispinulatum* isolates inferred by neighbor-joining (NJ) method with model of Kimura 2-parameter based on sequences of intergenic spacer rDNA (IGS rDNA). Bootstrap values (in %) above 50% from 1000 pseudoreplicates are shown for the NJ analyses. Scale bar indicates an evolutionary distance of Kimura 2-parameter methods.

Pairwise comparisons showed interspecific genetic variations of 54.6–56.8% between* O. dentatum* and* O. quadrispinulatum* isolates and intraspecific sequence differences of 0–1.3% and 0.2–15.1% for* O. dentatum* and* O. quadrispinulatum*, respectively. The phylogenetic relationships among* O. dentatum*,* O. quadrispinulatum,* and other known species were reconstructed using NJ analysis (Figure 2). From the NJ tree, two main clades were observed. All* Oesophagostomum* isolates were grouped in a sister clade, including* Trichostrongylus colubriformis* and* Marshallagia marshalli*, which suggested their close relationships with the two* Oesophagostomum* spp. Within the cluster of the two* Oesophagostomum* species, all* O. dentatum* isolates clustered together in one clade, whereas the* O. quadrispinulatum* isolates from different locations grouped into two distinct clusters. These results indicated the complicated genetic structure of* O. quadrispinulatum*.

The IGS is the most rapidly evolving region of rDNA, and the number and organization of the internal repeats are species-specific and often vary among populations, individuals, and even within a single cell [29]. These variations in the structures of repetitive regions are common in many taxa and have been widely used in phylogenetic analysis and to quantify gene flow between populations [29, 30]. Analyses of inter- and intragenetic variations in the IGS rDNA sequences of two species of pig nodule worms showed that the sequence differences between* O. quadrispinulatum* isolates were larger than those between* O. dentatum* isolates, and phylogenetic analysis revealed two subclusters within a clade of* O. quadrispinulatum*. These results suggested that different genotypes or subspecies might exist in* O. quadrispinulatum*.

## 4. Conclusions

This study is the first to report the 28S-18S IGS rDNA sequences of* O. dentatum* and* O. quadrispinulatum* from different geographical locations in China. Genetic analysis revealed the sequence annotations and organizations of these sequences and demonstrated that these regions were polymorphic and contained direct and inverted repeats. Different types and numbers of repeats and IGS rDNA structures could serve as potential markers for differentiating these two species of pig nodule worms.

## Figures and Tables

**Table 1:** Geographical origins, sample codes, and GenBank accession numbers of *Oesophagostomum dentatum* and *O. quadrispinulatum* samples used in the present study.

Species	Sample code	Geographical origin	Accession number
*O. dentatum *	ODHN1	Hunan (Huihua)	KC991159
	ODHN2	Hunan (Huihua)	KC991160
	ODYJ1	Guangdong (Yangjiang)	KC991161
	ODYJ2	Guangdong (Yangjiang)	KC991162
	ODYJ3	Guangdong (Yangjiang)	KC991163
	ODYJ4	Guangdong (Yangjiang)	KC991164
	ODYJ5	Guangdong (Yangjiang)	KC991165
	ODYC1	Chongqing (Yongzhou)	KC991166
	ODYC2	Chongqing (Yongzhou)	KC991167
	ODYC3	Chongqing (Yongzhou)	KC991168
	ODYC4	Chongqing (Yongzhou)	KC991169
	ODYC5	Chongqing (Yongzhou)	KC991170
	ODHLJ1	Heilongjiang (Jiaxing)	KC991171
	ODYB1	Chongqing (Yubei)	KC991172

*O. quadrispinulatum *	OQHN1	Hunan (Huihua)	KC991173
	OQRC1	Chongqing (Rongchang)	KC991174
	OQYC1	Chongqing (Yongchuan)	KC991175
	OQYC2	Chongqing (Yongchuan)	KC991176
	OQYC3	Chongqing (Yongchuan)	KC991177
	OQYC4	Chongqing (Yongchuan)	KC991178
	OQYC5	Chongqing (Yongchuan)	KC991179
	OQYC6	Chongqing (Yongchuan)	KC991180
	OQYC7	Chongqing (Yongchuan)	KC991181
	OQYC8	Chongqing (Yongchuan)	KC991182
	OQYC9	Chongqing (Yongchuan)	KC991183
	OQYC10	Chongqing (Yongchuan)	KC991184

*Trichostrongylus colubriformis *	—	Iran	HQ389237

*Marshallagia marshalli *	—	Iran	HQ389236

*Haemonchus contortus *	—	Iran	HQ389234

*Cylicostephanus minutus *	—	—	HM142941

*Coronocyclus coronatus *	—	—	HM142939
